# Characterization of the ligand-binding properties of odorant-binding protein 38 from *Riptortus pedestris* when interacting with soybean volatiles

**DOI:** 10.3389/fphys.2024.1475489

**Published:** 2025-01-06

**Authors:** Jianglong Guo, Panjing Liu, Xiaofang Zhang, Jingjie An, Yaofa Li, Tao Zhang, Zhanlin Gao

**Affiliations:** Plant Protection Institute, Hebei Academy of Agriculture and Forestry Sciences, Key Laboratory of Integrated Pest Management on Crops in Northern Region of North China, Ministry of Agriculture and Rural Affairs, IPM Innovation Center of Hebei Province, International Science and Technology Joint Research Center on IPM of Hebei Province, Baoding, China

**Keywords:** Riptortus pedestris, OBPs, soybean volatiles, fluorescence competitive binding, molecular docking

## Abstract

**Background:**

*Riptortus pedestris* (Fabricius) (Hemiptera: Alydidae) is a major soybean pest throughout East Asia that relies on its advanced olfactory system for the perception of plant-derived volatile compounds and aggregation pheromones for conspecific and host plant localization. Odorant binding proteins (OBPs) facilitate the transport of odorant compounds across the sensillum lymph within the insect olfactory system, enabling their interaction with odorant receptors (ORs).

**Methods:**

Real-time quantitative PCR (qRT-PCR) analyses, fluorescence-based competitive binding assays, and molecular docking analyses were applied to assess the expression and ligand-binding properties of OBP38 from *R. peddestris*.

**Results:**

The qRT-PCR analyses revealed high levels of *RpedOBP38* expression in the antennae without any apparent sex bias, and it was also highly expressed in the adult stage. Recombinant RpedOBP38 was prepared by expressing it in *E. coli* BL21 (DE3) followed by its purification with a Ni-chelating affinity column. RpedOBP38 was found to bind most strongly to trans-2-decenal (Ki = 7.440) and trans-2-nonenal (Ki = 10.973), followed by β-pinene, (+) -4-terpineol, carvacrol, methyl salicylate, and (-)-carvone. The 3D structure of RpedOBP38 contains six α-helices and three interlocked disulfide bridges comprising a stable hydrophobic binding pocket. In a final series of molecular docking analyses, several polar (e.g., His 94, Glu97) and nonpolar (e.g., Leu29, Ile59) residues were found to be involved in RpedOBP38-ligand binding.

**Conclusion:**

These data support a role for RpedOBP38 in the perception of volatiles derived from host plants, providing important insight into the mechanisms that govern olfactory recognition in *R. pedestris*, thereby informing the development of ecologically friendly approaches to managing *R. pedestris* infestations.

## 1 Introduction

The ability of insects to perceive pheromones, host-derived odorants, and the wide array of other peripheral chemical signals present in their surrounding environment is dependent on a complex olfactory system that ultimately shapes key physiological processes such as foraging, mating, and oviposition (Martin et al., 2011; Leal, 2013). The ability to accurately recognize and decipher these signals is thus vital for the ability of insects to survive and reproduce. Hydrophobic chemicals need to successfully penetrate the olfactory sensilla and the hydrophilic sensillum lymph in order to access the odorant receptors (ORs) present on sensory neuron surfaces, thereby triggering downstream signal transduction (Li et al., 2015; Zhou et al., 2022). To facilitate this process, specialized supporting cells produce odorant-binding proteins (OBPs), which are secreted into the olfactory sensillum lymph and play a vital role in the process of insect odorant reception (Leal, 2013; Paula et al., 2018). OBPs can selectively bind, solubilize, and transport odorant molecules as they diffuse into the sensillum lymph, thereby enabling the activation of ORs and associated downstream signaling pathways (Leal, 2013; Liu et al., 2023). Given the importance of OBPs during this initial stage of odorant reception, they hold great promise as molecular targets for pest control efforts and the development of superior integrated pest management (IPM) strategies (Zhou et al., 2010; Venthur and Zhou, 2018).

The OBPs produced by insects are low-molecular-weight (12–20 kDa) proteins approximately 100–200 amino acids in length that are water soluble and typically feature a ∼20 amino acid N-terminal signal peptide sequence (Ahmed et al., 2017; Li J. B. et al., 2022; Zeng et al., 2019). The 3D structures of classical OBPs are stabilized by three disulfide bridges formed by six conserved cysteine residues (Leal et al., 1999; Scaloni et al., 1999; Pelosi et al., 2014). The patterns of conserved cysteines have also been used to define four other classes of OBPs, including “Dimer” OBPs with two typical cysteines, “Minus-C” OBPs that lack 1-2 cysteines, “Plus-C″ OBPs with 2-3 extra cysteines, and “Atypical” OBPs with a long, atypical C-terminal domain (Zhou et al., 2010; Spinelli et al., 2012; Manoharan et al., 2013; Venthur et al., 2014; Zeng et al., 2019). Identified in 1981, the first characterized OBP in insects was found to be exclusively expressed in *Antberaea polypbemus* antennae, enabling male moths to detect a particular sex pheromone (*trans*-6, *cis*-11-hexadecadienyl acetate) such that they were able to locate conspecific females to engage in mating (Vogt and Riddiford, 1981). Advances in molecular biology and transcriptomic technologies have fueled the identification of a growing number of genes encoding OBPs in many orders of insects, including Coleoptera (e.g., 39 OBPs in *Phyllotreta striolata*, Xiao et al., 2023), Hemiptera (e.g., 49 OBPs in *Riptortus pedestris*, Li L. L. et al. (2022)), Diptera (e.g., 28 OBPs in *Liriomyza trifolii*, Zhang et al. (2022)), Lepidoptera (e.g., 31 OBPs in *Chilo sacchariphagus*, Liu et al. (2021a)), Hymenoptera (e.g., 21 OBPs in *Apis mellifera*, Forêt and Maleszka (2006)), Orthoptera (e.g., 22 OBPs in *Locusta migratoria*, Pelosi et al. (2018)). Experimental efforts have revealed that OBPs which are primarily expressed in the antennae of certain insects are capable of interacting with specific chemical ligands including host volatiles and pheromones (Zhang et al., 2020a; Rihani et al., 2021). AlepOBP6, for instance, is predominantly expressed in the antennae of male *Athetis lepigone* individuals and can recognize both maize-derived volatile compounds and sex hormones produced by conspecific females (Li J. B. et al., 2022). In *Hippodamia variegate*, both males and females exhibit high levels of HvarOBP5 expression in their antennae, thus enabling the perception of plant and prey-derived volatiles (Tang et al., 2023). The behavioral responses of *Eupeodes corolla* to the aphid alarm pheromone (E)-β-farnesene have been shown to be regulated by EcorOBP15 (Wang et al., 2022). Several OBPs have also been demonstrated to be expressed in other organs with or without primary chemosensory functions, including mouthpart palps (Pregitzer et al., 2018), labella (Sparks et al., 2014), legs (Hull et al., 2014), thorax (Zhang et al., 2018), and reproductive organs (Sun et al., 2012). These OBPs can facilitate a range of physiological functions including the recognition of taste compounds, the solubilization of nutrients, and the augmentation of resistance against insecticides (Pelosi et al., 2018).

*Riptortus pedestris* (Fabricius) (Hemiptera: Alydidae), known as the bean bug, is a serious agricultural pest species that is widely distributed throughout China, Japan, Korea, and other nations in East Asia (Jung and Lee, 2018; Jin et al., 2022). *R. pedestris* is a polyphagous pest species, feeding on over 30 different plants across 13 families (including Gramineae, Cruciferae, and other crop families), although they exhibit a particular preference for soybeans and other leguminous plants (Mainali et al., 2014; Ahn et al., 2020). Large numbers of these bean bugs typically infest soybean fields in the late flowering or early pod-growing stages and persistently feed on and damage these plants until harvest time (Endo et al., 2011). Soybean leaves, stems, pods, and flowers can be damaged by both *R. pedestris* adults and nymphs through their piercing and sucking behaviors, resulting in leaf rolling, stunted growth, and seed pods that are shriveled or empty, culminating in serious reductions in soybean quality and yield (Ahn et al., 2020). *R. pedestris*-associated soybean damage has recently emerged as a particularly serious problem in the Huang-Huai-Hai region of China (Li L. L. et al., 2022). Soybean plants in this region often suffer from the staygreen phenomenon that can be caused by *R. pedestris* feeding, which results in leaves that remain green, shriveled pods, and maturity stage seed abortion in soybean plants (Li et al., 2019; Dong et al., 2022). The control of *R. pedestris* has traditionally been achieved through the application of pyrethroids or other broad-spectrum insecticides (Gao et al., 2019; Guo et al., 2023). Such insecticide-based management practices, however, entail many potentially serious issues including environmental pollution, elevated levels of insecticide resistance, and inadequate efficacy owing to the highly mobile nature of these insects and their behavioral avoidance of insecticides (Bae et al., 2019; Zhu et al., 2022). There is thus a pressing need to develop new, ecologically friendly olfaction-based strategies for the control of *R. pedestris* infestations.

*R. pedestris* rely on their highly-developed antennae harboring abundant sensilla to detect both adult male-derived aggregation pheromone and host plant-derived volatiles, thus facilitating conspecific and host location efforts (Leal et al., 1995; Kim et al., 2016; Roh et al., 2021; Song et al., 2022). Li J. B. et al. (2022) previously analyzed the *R. pedestris* genome and identified 49 candidate RpedOBPs, including RpedOBP38, which exhibited high levels of expression in the antennae. The specific involvement of RpedOBP38 in the detection of host volatiles or other chemical signals, however, has yet to be documented. Accordingly, this study was devised to clarify the olfactory functions of RpedOBP38. To that end, the sequence of the *RpedOBP38* gene was initially analyzed, after which *RpedOBP38* expression was analyzed across a variety of tissues and developmental stages via real-time quantitative PCR (qRT-PCR). The binding affinity of RpedOBP38 for 36 volatiles (including 11 green leaf volatiles, 11 soybean volatiles, 10 volatiles associated with repellent activity, and 4 aggregation pheromone compounds) was characterized through a fluorescence binding assay. Lastly, homology modeling and molecular docking approaches were used to characterize the binding sites and key amino acids related to the ligand binding activity of RpedOBP38. Together, the results of these analyses provide a robust evidence base for the further molecular characterization of the mechanisms governing olfactory recognition in *R. pedestris*, thus supporting efforts to improve the integrated management of this economically significant pest species.

## 2 Materials and methods

### 2.1 Insect rearing and tissue collection

*R. pedestris* specimens were captured in July-August 2019 from soybean fields in Shijiazhuang, Hebei province, China. Adults and nymphs were reared as in prior reports (Guo et al., 2023). Briefly, these insects were housed at 26°C ± 1°C under 60% ± 5% relative humidity (RH) with a 16 h: 8 h (L:D) photoperiod in cages, and were fed dried seeds (variety Jidou 12) and soybean seedlings that were replaced every 5–7 days. Based on the study of Li L. L. et al. (2022), 3-day-old virgin male and female adults were processed to collect antennae (40 pairs), heads without antennae (from 10 individuals), thoraxes (from 4 individuals), abdomens (from 3 individuals), wings (from 40 individuals), and legs (from 20 individuals). In addition, antennae were collected from 2nd (200 pairs), 3rd (120 pairs), 4th (60 pairs), and 5th (60 pairs) instar nymphs, after which they were snap-frozen with liquid nitrogen and stored at −80°C.

### 2.2 Total RNA extraction and preparation

TRIzol (TransGen, China) was used to extract RNA according to the manufacturer’s instructions, the quality of which was analyzed via 1.0% agarose gel electrophoresis and spectrophotometry with a NanoDrop™ 2000 instrument (Thermo Fisher Scientific, United States). Next, 1 μg of the extracted RNA was processed with All-in-One First-Strand cDNA Synthesis SuperMix (TransGen), and the resultant cDNA was stored at −20°C.

### 2.3 Sequence alignment and phylogenetic analyses

The SignalP 6.0 server (https://services.healthtech.dtu.dk/services/SignalP-6.0/) was used for signal peptide prediction, while ClustalX 2.0 was used for multiple alignment of the RpedOBP38 protein sequence and those of other Hemiptera OBPs, with GeneDoc (http://nrbsc.org/gfx/genedoc) being used for result visualization. The amino acid sequences of other hemipteran species were downloaded by accessing the NCBI website. MEGA7 was used to construct a phylogenetic tree with the neighbor-joining method and bootstrap testing (1,000 replicates). The Poisson correction method was employed when calculating evolutionary distance.

### 2.4 *RpedOBP38* expression profiles

*RpedOBP38* expression was validated via qRT-PCR with an ABI QuantStudio6 Q6 Real-Time PCR System (Applied Biosystems, CA, United States) using primers designed with Premier 6 and prepared by Sangon Biotech Co., Ltd (Beijing, China) (Supplementary Table S1). Individual 20 μL reactions comprised 1 μL of cDNA, 0.6 μL each of F/R primers (10 μM), 10 μL of 2 × FastFire qPCR PreMix (TianGen Biotech, Beijing, China), and 7.8 μL of ddH_2_O. Reaction settings were: 94°C for 30 s; 40 cycles of 94°C for 5 s, 55°C for 15 s, and 72°C for 10 s. Relative *RpedOBP38* expression was assessed with the 2^-△△Ct^ method, using *EF1* and *Actin* as reference genes (Wang et al., 2023). Three independent biological replicates were analyzed per sample.

### 2.5 Recombinant plasmid construction

The *RpedOBP38* open reading frame (ORF) lacking a signal peptide sequence was PCR amplified with TransStart^®^ FastPfu PCR SuperMix (TransGen Biotech). Primers used to construct an RpedOBP38 expression vector were as follows: Forward: 5′-GAT​GAG​GCG​AAA​CAG​ATG-3′, Reverse: 5′-TCA​CTG​TAG​ATC​TTC​AGT​TCC-3’. Amplification settings were as follows: 95°C for 1 min; 35 cycles of 95°C for 20 s, 55°C for 20 s, and 72°C for 1 min; 72°C for 5 min. The products of PCR amplification were ligated into the pEASY-Blunt E1 vector (TransGen Biotech) and transformed into *E. coli Trans*-T1. Sangon Biotech then sequenced and confirmed the amplified gene products, and positive recombinant pEASY-Blunt E1-RpedOBP38 plasmids were obtained for further use.

### 2.6 Recombinant RpedOBP38 purification

After transforming *E. coli* BL21 (DE3) with recombinant RpedOBP38 expression vectors, positive clones were isolated and used to initiate cultures in LB broth containing 50 μg/mL ampicillin that were incubated at 37°C and 220 rpm. When the OD_600_ reached 0.6, 1 mM of isopropyl *β*-D-thiogalactoside (IPTG) was added and bacteria were incubated under the same conditions for a further 6 h. Cells were then centrifuged (8,000 xg, 4°C) and resuspended in 20 mL of PBS (pH 7.0). Cells were then ultrasonically disrupted, and homogenates were centrifuged (14,000 rpm, 20 min, 4°C). The supernatants were then assessed via 12% SDS-PAGE separation. Target proteins from the supernatant fractions were applied to a Ni-chelating affinity column (GE, United States), which was subsequently equilibrated with 100 mM NaCl, 20 mM Tris-HCl, pH 7.9, and eluted using an ascending imidazole concentration series (50, 100, 150 and 200 mM). Dialysis was used to desalt the eluent, and target protein size and purity were assessed via SDS-PAGE. Recombinant protein concentrations were measured via Bradford assay.

### 2.7 Fluorescence competitive binding assay

Recombinant RpedOBP38 binding to putative chemical ligands was characterized with a microplate reader (BioTek Synergy H1, United States). Fluorescence intensity values at the excitation wavelength of 337 nm and a maximum fluorescence emission wavelength of 450 nm were plotted against the free concentration of ligand for the measurement of dissociation constants, selecting candidate ligands from among 36 volatile compounds that included 11 green leaf volatiles (Chen et al., 2018; Guo and Wang, 2019; Cheng et al., 2020; Hong et al., 2022; Tang et al., 2023; Zhu et al., 2023), 11 soybean volatiles (Wang et al., 2019a; Zhu et al., 2022), 10 repellent activity volatiles (Zhang et al., 2013; Zhang et al., 2014), and 4 aggregation pheromone compounds (Leal et al., 1995; Yasuda et al., 2007). HPLC-grade methanol was used for the dissolution of the probe N-phenyl-1-naphthylamine (1-NPN) and all ligands. The ability of RpedOBP38 to bind 1-NPN was assessed by using 10 mM PBS (pH 7.4) to prepare a 2 μM purified protein solution, titrating with 1 mM 1-NPN in methanol to prepare final concentrations from 2–20 μM. RpedOBP38 binding to each ligand was evaluated in a solution consisting of 2 μM purified protein and 1-NPN, followed by titration through the addition of ligands until no further decrease in the fluorescence intensity was observed. Ligands were independently replicated three times, and dissociation constants for each ligand were measured as follows: Ki = [IC50]/(1 + [1-NPN]/K_1-NPN_), where IC50 denotes the ligand concentration when the fluorescence intensity is half of the initial value [1-NPN] is the free 1-NPN concentration, and K_1-NPN_ is the dissociation constant for the RpedOBP38/1-NPN complex (Campanacci et al., 2001). Based on the study of Cui et al. (2018), the strength of binding affinity could be indicated by Ki value, including very strong (Ki < 6 μM), strong (6 µM ≤ Ki < 22 µM), moderate (22 µM ≤ Ki < 40 µM) and weak (Ki > 40 µM).

### 2.8 Homology modelling and molecular docking analyses

RpedOBP38 tertiary structure modeling was performed with the I-TASSER server (Zheng et al., 2021), due to the <30% homology with the protein sequences in the Swiss-model server. The RpedOBP38 amino acid sequence was utilized as an input, utilizing the 10 template proteins exhibiting the highest sequence identity for the purposes of modeling (Supplementary Table S2). C-score values were used to choose the best model from among the top 5 (Supplementary Table S3), with C-scores generally falling in the [-5, 2] range, and higher scores being indicative of greater model confidence. For the chosen ligands, the PubChem database (https://pubchem.ncbi.nlm.nih.gov/) was accessed to download 3D structures that were subsequently converted into the mol2 format with Open Babel GUI v.3.1.1 (O’Boyle et al., 2011). Molecular docking analyses of the interactions between RpedOBP38 and seven ligands were performed with AutoDock Vina (v.1.1.2) (Trott and Olson, 2010) using default parameters. PyMOL v.2.0 (Schrödinger, LLC) was used for the visualization of the molecular docking results, and interaction forces were examined with PLIP (https://plip-tool.biotec.tu-dresden.de/plipweb/plip/index).

### 2.9 Statistical analyses

*RpedOBP38* expression was analyzed across various *R. pedestris* tissues and developmental stages using one-way ANOVA with Tukey’s multiple comparison test. *p* < 0.05 was selected as the cut-off for significance, and SPSS 20.0 (IBM 2011) was used for all statistical analyses, while GraphPad Prism 8.0 was used for figure generation.

## 3 Results

### 3.1 RpedOBP38 sequence analyses

**FIGURE 1:**
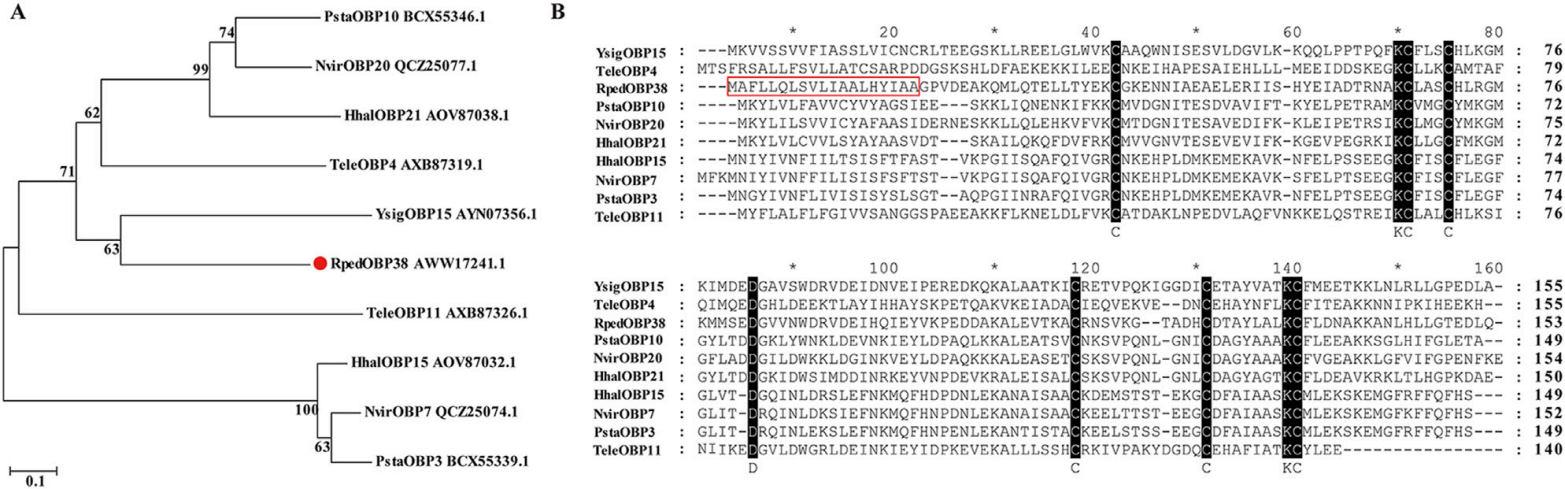
RpedOBP38 sequence characteristics. **(A)** Odorant-binding proteins (OBPs) from *Riptortus pedestris* and other hemipteran species were used to construct a phylogenetic tree. **(B)** RpedOBP38 alignment to OBP sequences from other hemipteran species. The signal peptide sequence is marked with a red box. Hhal: *Halyomorpha halys*, Nvir: *Nezara viridula*, Psta: *Plautia stali*, Tele: *Tropidothorax elegans*, Ysig: *Yemma signatus.*

*RpedOBP38* cDNA sequences were downloaded from the *R. pedestris* genome (Li J. B. et al., 2022). The *RpedOBP38* ORF was found to consist of 462 bp encoding a 153-amino-acid (aa) protein, with a 19-aa N-terminal signal peptide. This protein had a predicted molecular mass of 15.08 kDa and a predicted isoelectric point of 5.20. BLASTp similarity analyses revealed some level of sequence identity with OBPs from other Hemiptera species, including YsigOBP15 from *Yemma signatus* (43.42%), PstaOBP3 from *Plautia stali* (38.78%), HhalOBP15 from *Halyomorpha halys* (37.50%), TeleOBP5 from *Tropidothorax elegans* (36.23%), and NvirOBP20 from *Nezara viridula* (35.71%). Phylogenetic tree analyses revealed the clustering of RpedOBP38 and YsigOBP15 from *Y. signatus* (Figure 1A). Sequence alignment also revealed the presence of six conserved cysteine residues within RpedOBP38 (Figure 1B), consistent with its classification within the classical OBP family.

### 3.2 Evaluation of *RpedOBP38* expression patterns

**FIGURE 2:**
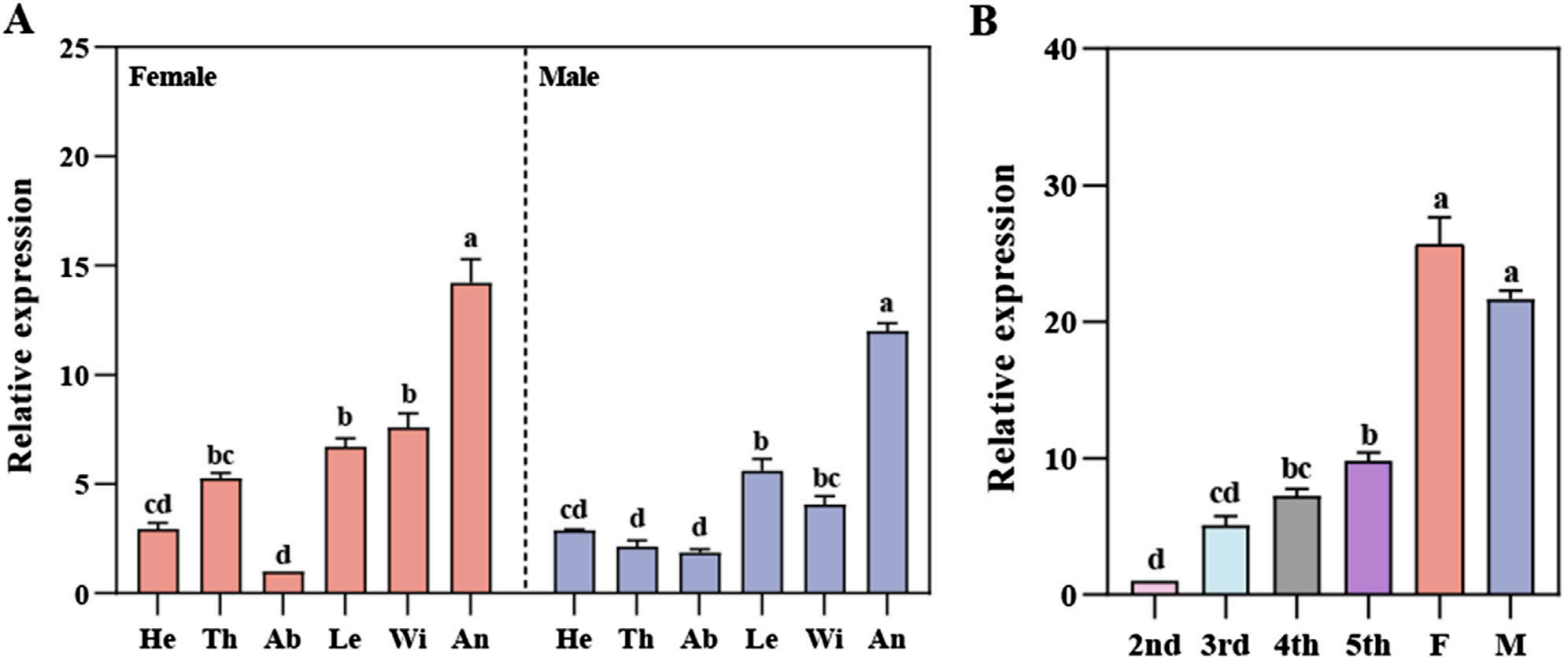
Relative *RpedOBP38* expression analyses. **(A)** qRT-PCR analyses of *RpedOBP38* mRNA levels in various tissues. He: heads, Th: thoraxes, Ab: abdomens, Le: legs, Wi: wings, An: antenna. **(B)** qRT-PCR analyses of *RpedOBP38* mRNA levels in the antennae of *Riptortus pedestris* at different developmental stages. F: female, M: male. Significant differences are indicated by different lowercase letters (*p* < 0.05; Tukey’s HSD test).

*RpedOBP38* expression across tissues and developmental stages was next characterized by qPCR, revealing significant differences in *RpedOBP38* among tissues in both female (*F*_*5, 12*_ = 65.68, *P* < 0.001) and male (*F*_*5, 12*_ = 129.09, *P* < 0.001) adults, with the highest expression levels in the antennae of adult females and males, respectively (Figure 2A). Antennae *RpedOBP38* expression levels rose with increasing developmental stages, with significant differences among stages (*F*_*5, 12*_ = 106.62, *P* < 0.001), and expression levels being highest in adult antennae. However, there was no significant sex difference for *RpedOBP38* expression levels (Figure 2B).

### 3.3 Characterization of RpedOBP38 binding to 1-NPN and candidate ligands

**FIGURE 3:**
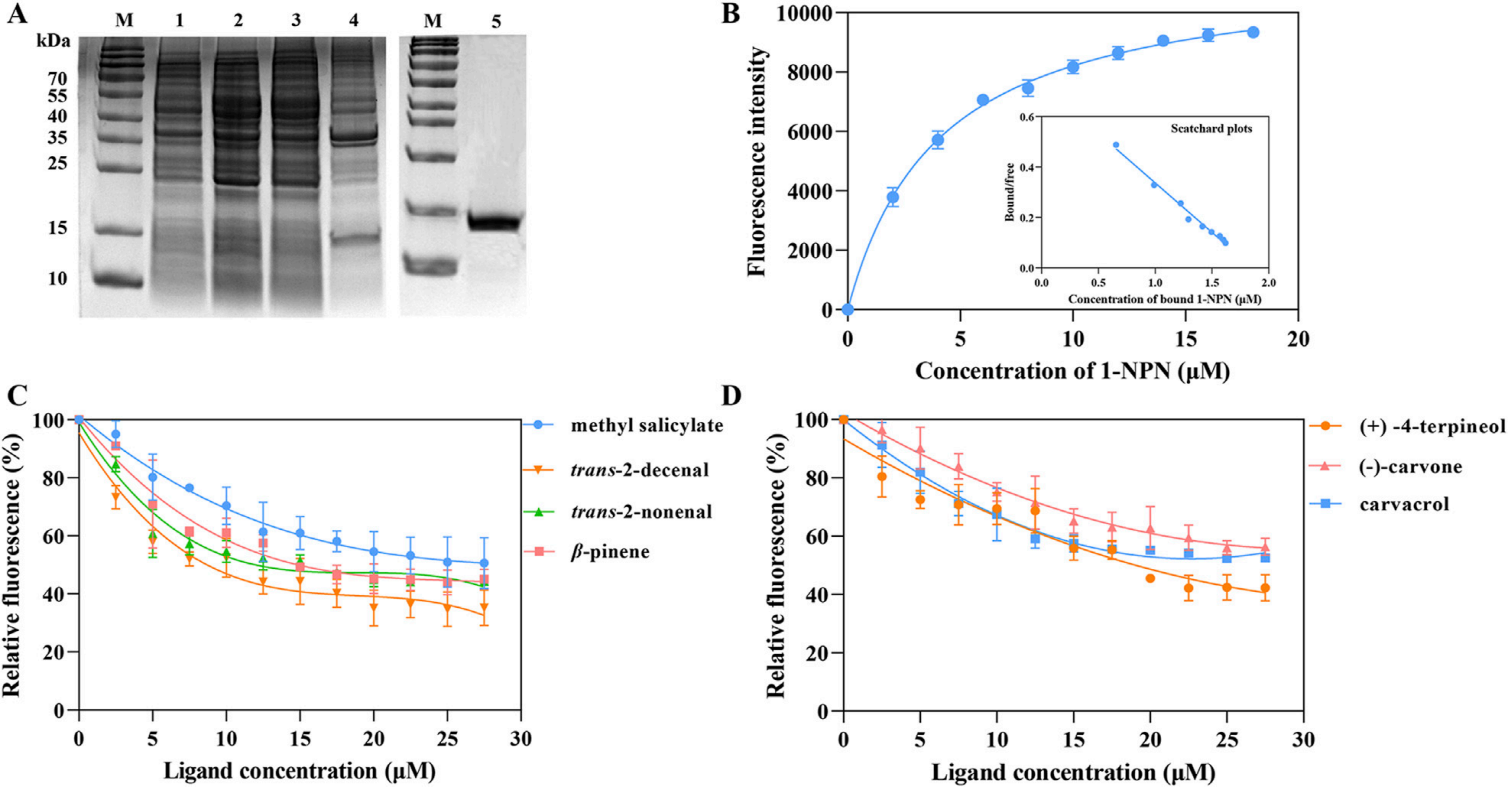
Characterization of RpedOBP38 ligand binding properties. **(A)** SDS-PAGE analyses pertaining to recombinant RpedOBP38 expression and purification. Lane 1: non-induced pEasy-Blunt E1-RpedOBP38; Lane 2: induced pEasy-Blunt E1-RpedOBP38; Lane 3: Supernatant of induced pEasy-Blunt E1-RpedOBP38; Lane 4: precipitation of induced pEasy-Blunt E1-RpedOBP38; Lane 5: purified RpedOBP38. **(B)** Binding curves and scatchard plots correspond to the interaction between the fluorescent probe 1-NPN and RpedOBP38. **(C, D)** Binding curves corresponding to interactions between RpedOBP38 and green leaf volatiles, soybean volatiles. **(C)** or repellent volatiles **(D)**.

After expressing recombinant RpedOBP38 in *E. coli* BL21 (DE3), it was purified, yielding a final recombinant RpedOBP38 concentration of 0.603 mg/mL. SDS-PAGE analyses confirmed a similar target protein size to the predicted size (Figure 3A). The ability of RpedOBP38 to bind 1-NPN was then assessed, revealing strong binding between RpedOBP38 and 1-NPN (dissociation constant [K_d_]): 4.059 μmol/L). Binding curve analyses and Scatchard plots revealed the presence of a single binding site, indicating that 1-NPN was a highly suitable probe for subsequent binding analyses (Figure 3B).

In total, 36 volatile compounds including 11 green leaf volatiles, 11 soybean volatiles, 10 volatiles associated with repellent activity, and 4 aggregation pheromone compounds were chosen for the evaluation of RpedOBP38 ligand binding. The resultant analyses demonstrated the ability of RpedOBP38 to strongly bind to the soybean volatiles *trans*-2-decenal (Ki = 7.440 μM), *trans*-2-nonenal (Ki = 10.973 μM) and methy salicylate (Ki = 21.065 μM) (Figure 3C; Table 1). It also exhibited strong or moderate binding to three volatiles associated with repellent activity ((+) -4-terpineol, Ki = 14.017 μM; carvacrol, Ki = 19.446 μM; (−)-carvone, Ki = 27.215 μM) (Figure 3D; Table 1). In contrast, it exhibited weak binding activity for the tested aggregation pheromone compounds (Ki > 40 μM) (Table 1).

**TABLE 1:** Binding affinities of all tested ligands to RpedOBP38.

Ligands	CAS number	IC50 (μmol/L)	Ki (μmol/L)
Green leaf volatiles			
*trans*-2-hexenal	6,728-26-3	>40	>40
octanal	124-13-0	>40	>40
nonanal	124-19-6	>40	>40
*β*-myrcene	123-35-3	>40	>40
*trans*-caryophyllene	87-44-5	>40	>40
*α*-pinene	13,877-91-3	>40	>40
geraniol	106-24-1	>40	>40
camphene	565-00-4	>40	>40
*β*-pinene	127-91-3	16.393 ± 0.261	12.315 ± 0.196
(+)-*α*-pinene	7,785-70-8	>40	>40
(−)-*α*-pinene	7,785-26-4	>40	>40
Soybean volatiles			
hexanal	66-25-1	>40	>40
1-hexanol	111-27-3	>40	>40
1-octen-3-ol	3,391-86-4	>40	>40
3-octanone	106-68-3	>40	>40
*cis*-3-hexen-1-ol	928-96-1	>40	>40
*cis*-3-hexenyl acetate	3,681-71-8	>40	>40
methyl salicylate	119-36-8	28.039 ± 5.938	21.065 ± 4.461
*trans*-2-hexenyl acetate	2,497-18-9	>40	>40
*trans*-2-octenal	2,548-87-0	>40	>40
*trans*-2-nonenal	18,829-56-6	14.606 ± 0.821	10.973 ± 0.617
*trans*-2-decenal	3,913-81-3	9.904 ± 0.970	7.440 ± 0.729
Volatiles with repellent activity			
eugenol	97-53-0	>40	>40
isoeugenol	97-54-1	>40	>40
(−) -4-terpineol	20,126-76-5	>40	>40
(+) -4-terpineol	2,438-10-0	18.658 ± 0.428	14.017 ± 0.321
*γ*-terpinene	99-85-4	>40	>40
cineole	470-82-6	>40	>40
*α*-terpinene	99-86-5	>40	>40
(−)-carvone	6,485-40-1	36.226 ± 5.169	27.215 ± 3.883
(+)-carvone	2,244-16-8	>40	>40
carvacrol	499-75-2	25.898 ± 0.443	19.456 ± 0.333
Pheromone compounds			
*trans*-2-hexenyl hexanoate	53,398-86-0	>40	>40
(E)-2-hexenyl (Z)-3-hexenoate (E2-6:Z3Hex)	53,398-87-1	>40	>40
(E)-2-hexenyl (Z)-2-hexenoate (E2-6:E2Hex)	54,845-28-2	>40	>40
myristyl isobutyrate	167,871-30-9	>40	>40

‘Ki > 40 μM’ means that the binding ability of RpedOBP38 recombinant protein to this ligand was considered weak.

### 3.4 Homology modeling and molecular docking analyses

**FIGURE 4:**
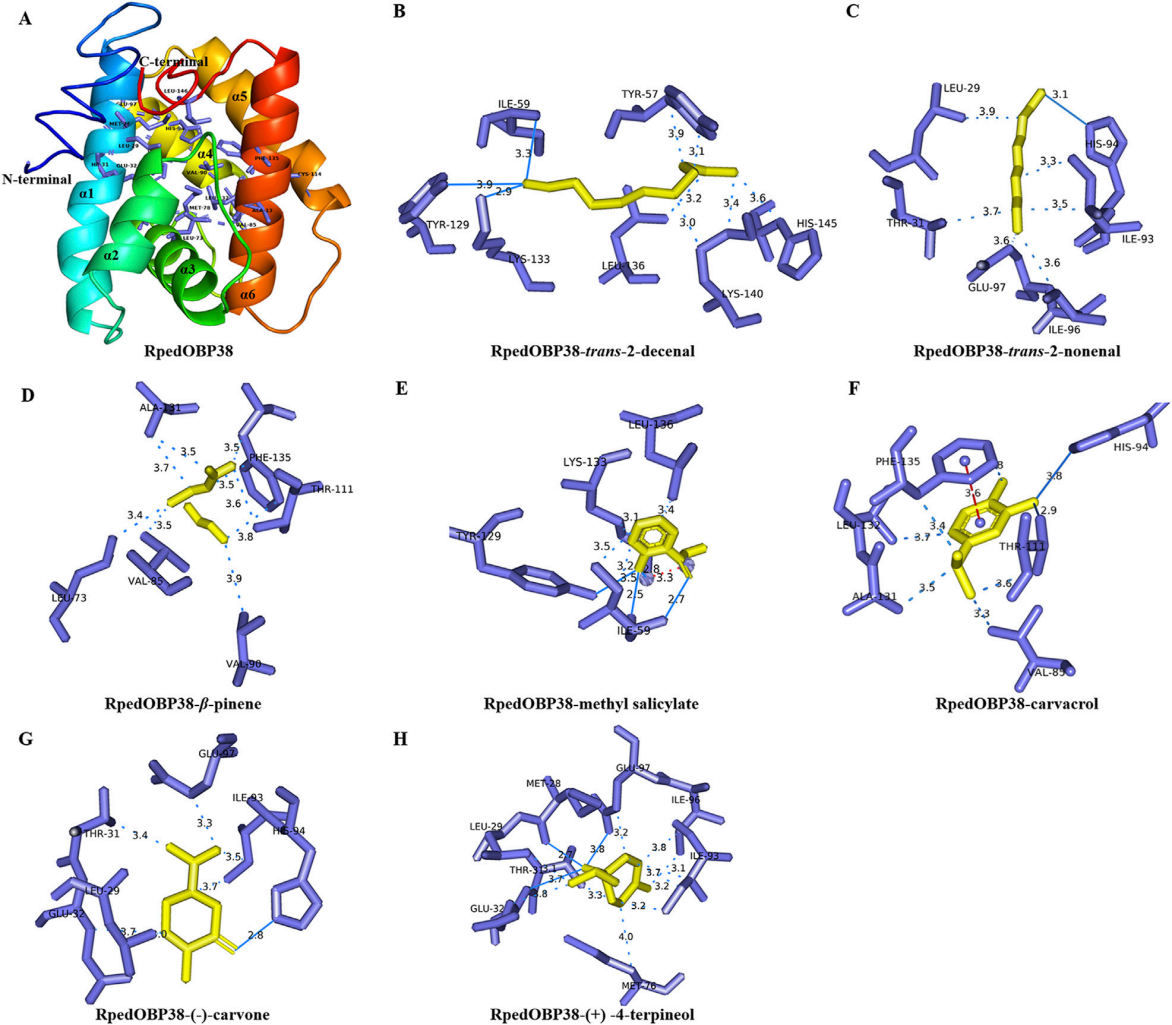
Molecular docking of ligands within the putative RpedOBP38 ligand binding pocket. **(A)** A structural model of RpedOBP38. The indicated amino acid residues correspond to key residues within the predicted RpedOBP38 pocket. **(B–H)** Molecular docking analyses for interactions between RpedOBP38 and *trans*-2-decenal **(B)**, *trans*-2-nonenal **(C)**, *β*-pinene **(D)**, methyl salicylate **(E)**, carvacrol **(F)** (−)-carvone **(G)**, and (+) -4-terpineol **(H)**. Hydrogen bonds are indicated with blue lines, while hydrophobic interactions are denoted using blue dashed lines, and π-stacking is represented using red dashed lines.

When the I-TASSER server was used to construct 3D models of the structure of RpedOBP38, the first model among the top five generated models exhibited the highest C-score of −1.13 (Supplementary Table S3). This predicted RpedOBP38 model contained six *α*-helices designated *α*1 (Pro21-Glu42), *α*2 (Glu47-Ser55), *α*3 (Cys67-Gly75), *α*4 (Trp87-Glu97), *α*5 (Pro101-Ala113), and *α*6 (His124-Ala142) that were folded around a hydrophobic cavity. It also harbored three interlocking disulfide bridges formed by links between Cys39 in *α*1 and Cys71 in *α*3, Cys67 in *α*3 and Cys125 in *α*6, and Cys114 in *α*5 and Cys134 in *α*6, providing further stability to the hydrophobic structure of this protein (Figure 4A).

Based on the fluorescence competitive binding assays performed above, *β*-pinene, methyl salicylate, *trans*-2-nonenal, *trans*-2-decenal (+) -4-terpineol (−)-carvone, and carvacrol were chosen as target ligands for molecular docking analyses. All seven of these ligands exhibited negative binding energy values when interacting with RpedOBP3 ranging from −5.71 to −4.20 (Table 2). Hydrogen bonds, hydrophobic interactions, and π-stacking were all found to contribute to these RpedOBP38-ligand binding interactions (Figures 4B–H). Both polar (e.g., Lys133, Glu97, His 94, Thr31) and nonpolar (e.g., Ile59, Leu136, Val85, Phe135) residues within the hydrophobic RpedOBP38 cavity were found to contribute to intermolecular binding interactions. Some of these amino acid residues were found to bind to multiple ligands, including 10 (Glu32, Ile59, Val85, Ile96, Thr111, Tyr129, Ala131, Lys133, Phe135, and Leu136) that were able to bind to three ligands, and 5 (Leu29, Thr31, Ile93, His94, and Glu97) that were able to bind to three ligands (Table 2).

**TABLE 2:** Prediction of key amino acid residues involved in the docking of RpedOBP38 to different ligands.

Ligands	Binding energy (kcal/mol)	Closer contact interacting residues
*β*-pinene	−5.07	LEU73, VAL85, VAL90, THR111, ALA131, PHE135
methyl salicylate	−5.42	**ILE59**, **TYR129**, **LYS133**, *LYS133*, LEU136
*trans*-2-nonenal	−4.20	LEU29, THR31, ILE93, **HIS94**, ILE96, GLU97
*trans*-2-decenal	−5.17	TRY57, **ILE59**, **TYR129**, **LYS133**, LEU136, LYS140, HIS145
(+)-4-terpineol	−5.30	**MET28**, LEU29, THR31, **GLU32**, MET76, ILE93, ILE96, **GLU97**
(−)-carvone	−5.71	LEU29, THR31, GLU32, ILE93, **HIS94**, GLU97
carvacrol	−5.52	VAL85, **HIS94**, **THR111**, ALA131, LEU132, *PHE135*

Amino acids in bold font represent hydrogen bond, amino acids in italic represent *π*-stacking, and other amino acids represent hydrophobic interaction.

## 4 Discussion

OBPs have been identified across many insect species to date and have been confirmed to be integral to the recognition of exogenous chemical signals and the regulation of physiological activities (Venthur and Zhou, 2018). OBPs have been established as promising molecular targets when screening for odorous compounds with attractant or repellent properties, informing the development of push-pull pest control strategies (Zhang et al., 2021; Song et al., 2022; Zhu et al., 2022; Zhu et al., 2023). For instance, CquiOBP1 of *Culex quinquefasciatus* was used as a target to guide the successful synthesis of a blend of trimethylamine and nonanal through the combination of conventional and reverse chemical ecology methodological approaches (Leal et al., 2008). The ability of certain OBPs to bind to aphid alarm pheromone has also enabled the design and synthesis of novel (*E*)-*β*-farnesene analogs with repellent and insecticidal activity for *Acythosiphon pisum* (Sun et al., 2011). In light of the importance of OBPs and the rising demand for environmentally friendly approaches to managing pest species, OBPs have emerged as a research hotspot in the insect chemical ecology space.

Initial sequencing analyses performed in this study revealed that RpedOBP38 had 153 amino acids in length with a 19-aa N-terminal signal peptide and six conserved cysteine residues, consistent with its classification as a member of the classic OBP family (Pelosi et al., 2006; Brito et al., 2016). Phylogenetic analyses can be used to infer evolutionary relationships for particular genes across species, thereby informing functional analyses such that they have been widely used for characterizing insect OBPs (Chen et al., 2018; Zhang et al., 2022; Tang et al., 2023; Zhu et al., 2023). In this study, RpedOBP38 and YsigOBP15 from *Y. signatus* clustered together, suggesting their evolution from a shared ancestor and their potential for similar physiological functions. Analyzing the patterns of insect OBP expression across developmental stages and tissues is vital for the clarification of the physiological functions of these factors (Ju et al., 2014; Wang et al., 2019b; Li et al., 2020). In general, OBPs are likely to play a role in the recognition of chemical signals if they are expressed at high levels in antennae and other olfactory organs (Chen et al., 2018; Li et al., 2020; Hong et al., 2022; Zhang et al., 2022; Tang et al., 2023). Higher levels of *RpedOBP38* expression were noted in the antennae relative to other tissues in this study, with no significant difference between females and males. This result was in line with a prior report by Li L. L. et al (2022), indicating that *RpedOBP38* may play an important role in the recognition of host volatiles and/or aggregation pheromones by *R. pedestris*. Other groups have also reported similar outcomes. For instance, Huang et al. (2018) reported the specific expression of AipsOBP2 in *Agrotis ipsilon* antennae and found that it was capable of binding both host volatiles and sex pheromones. Cheng et al. (2020) additionally noted the strong binding of SmosOBP12, which was expressed at high levels in the antennae of female *Sitodiplosis mosellana*, to host volatiles derived from wheat including hexyl acetate and 3-hexanol. *R. pedestris* reportedly harbor many different olfactory sensors on their antennae (Kim et al., 2016), and are attracted to soybean-derived volatile compounds and aggregation pheromones released by conspecific males (Leal et al., 1995; Song et al., 2022). High levels of RpedOBP38 expression were also noted in the adult stage, suggesting its potential involvement as a mediator of soybean volatile and aggregation pheromone recognition in *R. pedestris*.

Given the role that OBPs play as carriers in the context of chemical communication in insects, there is a need to clarify the affinity of these compounds for exogenous organic factors including pheromones and host-derived odorants, thereby potentially offering insight into the structural features of cognate ligands to guide reverse chemical ecology studies (D'Onofrio et al., 2020). Fluorescence competitive binding have been established as a reliable means of assessing *in vitro* binding between OBPs and their ligands (He et al., 2019; D'Onofrio et al., 2020). This approach has been successfully implemented across various species of insects including *Diaphorina citri* (Liu et al., 2021b), *Liromyza trifolii* (Zhang et al., 2022), *R. pedestris* (Zhu et al., 2022), *Bradysia odoriphaga* (Zhu et al., 2023), and *Hippodamia variegate* (Tang et al., 2023). In this study, the ability of RpedOBP38 to bind to 11 green leaf volatiles, 11 soybean volatiles, 10 volatiles with repellent activity, and 4 aggregation pheromone compounds was assessed. In total, it was found to bind to three soybean volatiles (*trans*-2-decenal, *trans*-2-nonenal, methyl salicylate) and one green leaf volatile (*β*-pinene). Host plant volatiles have been shown to promote feeding, avoidance, oviposition, and a range of other behavioral responses (Anderson et al., 1993; Leal et al., 1994; Zhu et al., 2022). RpedOBP38 may thus play a role in the detection of soybean volatiles, although behavioral and RNA interference assays will be necessary to confirm this hypothesis. Zhu et al. (2022) previously demonstrated the ability of RpedOBP4 to bind other soybean volatiles including 1-hexanol and *trans*-2-hexenyl acetate, supporting the potential involvement of multiple OBPs in the process of host plant recognition in line with what has been reported by Li et al. (2017). RpedOBP38 was also able to bind less strongly to plant essential oil-derived volatiles with repellent activity ((+) -4-terpineol (−)-carvone, carvacrol) that exhibit high levels of repellency for various insect species (Quintana et al., 2009; Zhang et al., 2014). However, the binding affinity of RpedOBP38 for tested aggregation pheromones was low (Ki > 40 μM), suggesting that binding to these compounds may be primarily mediated by other chemosensory proteins including RpedCSP12 (Yin et al., 2023). Notably, RpedOBP38 exhibited distinct binding affinity levels for certain isomers as in the case of (+) -4-terpineol (Ki = 14.02 μM) and (−) -4-terpineol (Ki > 40 μM), or (−)-carvone (Ki = 27.22 μM) and (+)-carvone (Ki > 40 μM). Factors including carbon chain length, conformational changes, and structural features can thus likely shape RpedOBP38 binding affinity (Chen et al., 2018; Hong et al., 2022).

The physiological functions of a given protein are determined by its 3D structure, and insect OBPs generally harbor a hydrophobic cavity formed from multiple *α*-helices, with some of the amino acids therein facilitating interactions between these OBPs and their ligands (He et al., 2019; Zhang et al., 2020b; Yang et al., 2021; Zhu et al., 2023). Molecular modeling analyses performed herein revealed the presence of a hydrophobic binding pocket within RpedOBP38 that was stabilized by six *α*-helices and three interlocking disulfide bridges. This is consistent with similar reports for DcitOBP7 in *Diaphorina citri* (Liu et al., 2021a), and PyasOBP2 in *Pachyrhinus yasumatsui* (Hong et al., 2022), suggesting that they may engage in similar ligand-binding mechanisms. Molecular docking analyses revealed negative binding energy values for interactions between RpedOBP38 and seven analyzed ligands, implying strong protein-ligand interactions, consistent with the fluorescence competitive binding assay results. OBP-ligand binding is generally mediated by types of intermolecular forces including hydrogen bonds, van der Waals interactions, and hydrophobic interactions (Zhuang et al., 2014; Li et al., 2021; Hong et al., 2022). In this study, hydrogen bonds, hydrophobic interactions, and π-stacking were all found to shape RpedOBP38-ligand interactions, with molecular docking analyses also revealing the distribution of several polar (e.g., Lys133, Glu97, His 94, Thr31) and nonpolar (e.g., Ile59, Leu136, Val85, Phe135) residues within the RpedOBP38 hydrophobic pocking jointly contributing to such intermolecular binding. This aligns well with other reports for insect OBPs, including the Val114, Thr9, and Val111 residues in *Grapholita Molesta* OBP2 (Li et al., 2016), Tyr77, Ile41, Ala116, and Lys38 in *Apbid Sitobion* OBP9 (Ullah et al., 2020), Leu33, Phe8, Met76, IIe30, Tyr47, Asp29, and Lys120 in *R. pedestris* OBP4 (Zhu et al., 2022), and Lys43, His64, and Leu42 in *H, variegate* OBP5 (Tang et al., 2023). Some of these amino acids were found to be capable of binding to more than one ligand, including Leu29, Thr31, His94, Glu97, Ile59, and Lys133, in line with what has previously been described in both *Athetis lepigone* (Li L. L. et al., 2022) and *R. pedestris* (Zhu et al., 2022). These residues may thus be particularly important mediators of RpedOBP38-ligand binding, highlighting an opportunity for site-directed mutagenesis to validate this hypothesis in the future (Zhu et al., 2020).

In summary, these experiments revealed that RpedOBP38, which was highly expressed in the antennae of adult *R. pedestris,* is a classical OBP family member that clusters most closely with YsigOBP15 from *Y. signatus*. Fluorescence competitive binding analyses demonstrated the ability of RpedOBP38 to bind strongly to two soybean volatiles (*trans*-2-decenal, Ki = 7.440 μM; *trans*-2-nonenal, Ki = 10.973 μM; methyl salicylate, Ki = 21.065 μM) and to bind strongly or moderately to volatiles associated with repellent activity ((+) -4-terpineol, Ki = 14.017 μM; carvacrol, Ki = 19.456 μM; (−)-carvone, Ki = 27.215 μM). Through 3D modeling and molecular docking analyses, RpedOBP38 was found to harbor six *α*-helices that form a stable hydrophobic binding pocket, with the Leu29, Thr31, His94, Glu97, Ile59, and Lys133 amino acid residues all playing key roles in the ability of this OBP to bind its ligands. Together, these results offer further insight into the mechanisms that govern olfactory recognition in *R. pedestris*. In order to more deeply elucidate the function of RpedOBP38, future studies are planned to analyse the exact role of RpedOBP38 in the recognition of more green leaf volatiles and soybean volatiles using a combination of behavioural experiments, electrophysiological experiments, and RNA inference (Zhu et al., 2022). Furthermore, we attempt to use RpedOBP38 as a control target, devise ecologically friendly behavioural inhibitors to disrupt the feeding behavior of *R. pedestris* and thus improve the management of *R. pedestris* (Zhu et al., 2022).

## Data Availability

The datasets presented in this study can be found in online repositories. The names of the repository/repositories and accession number(s) can be found in the article/Supplementary Material.
