# Supplementary material for: Characterization of the ligand-binding properties of odorant-binding protein 38 from Riptortus pedestris when interacting with soybean volatiles
Source: Front Physiol. 2025 Jan 6;15:1475489. doi: 10.3389/fphys.2024.1475489 (PMC11743672; doi:10.3389/fphys.2024.1475489)
Supplement: Supplementary file 1 [file Table1.docx]

Supplementary Material

# Supplementary Tables

**Table S1 Primers used in relative expression analyses of *RpedOBP38*.**

| **Gene name** | **Primer sequence (5’–3’)** |
| --- | --- |
| *Actin* | F: AGTGGAGATGGCGTAACA |
|  | R: GTGCTTCAGGTGCTTCAA |
| *EF1* | F: TTGCCAACGGTTACACTC |
|  | R: CGCCAGACTTGATAGACTTA |
| *RpedOBP38* | F: GGCATCATGTCATCTTCGAGGAATG |
|  | R: TAGGGCTTTGGCATCATCTTCAGG |

**Table S2 Top ten threading templates used by I-TASSER.**

| **Rank** | **PDB Hit** | **Idenl** | **Iden2** | **Cov** | **Norm. Hit Z-core** |
| --- | --- | --- | --- | --- | --- |
| 1 | 7uo6A | 0.21 | 0.24 | 0.86 | 2.08 |
| 2 | 7xyqB | 0.10 | 0.14 | 0.81 | 1.61 |
| 3 | 3q8i | 0.21 | 0.20 | 0.79 | 1.93 |
| 4 | 7vw8 | 0.14 | 0.18 | 0.86 | 1.44 |
| 5 | 6qq4A | 0.25 | 0.22 | 0.78 | 2.10 |
| 6 | 7vw8 | 0.15 | 0.18 | 0.80 | 2.18 |
| 7 | 4inwA | 0.14 | 0.22 | 0.86 | 1.91 |
| 8 | 6qq4A | 0.25 | 0.22 | 0.78 | 1.74 |
| 9 | 7uo6A | 0.22 | 0.24 | 0.87 | 1.55 |
| 10 | 4pt1A | 0.17 | 0.20 | 0.82 | 3.13 |

Iden1, the percentage sequence identity of the templates in the threading aligned region with the sequence of RpedOBP38; Iden2, the percentage sequence identity of the whole template chains with query sequence; Cov, the coverage of the threading alignment and is equal to the number of aligned residues divided by the length of query protein; Norm. Z-score, the normalized Z-score of the threading alignments. Alignment with a Normalized Z-score >1 mean a good alignment and vice versa.

**Table S3 Top five final models predicted by 1-TASSER.**

| **Model** | **C-Score** | **Estimated TM-score** | **Estimated RMSD** |
| --- | --- | --- | --- |
| Model 1 | -1.13 | 0.57 ± 0.14 | 7.2±4.2Å |
| Model 2 | -2.56 |  |  |
| Model 3 | -2.10 |  |  |
| Model 4 | -2.68 |  |  |
| Model 5 | -1.25 |  |  |
